# Network analysis reveals dysregulated functional patterns in type II diabetic skin

**DOI:** 10.1038/s41598-022-10652-8

**Published:** 2022-04-27

**Authors:** Chunan Liu, Sudha Ram, Bonnie L. Hurwitz

**Affiliations:** 1 Department of Biosystems Engineering, BIO5 Institute, University of Arizona, Tucson, AZ 85721 USA; 2 Department of Management Information Systems, BIO5 Institute, University of Arizona, Tucson, AZ 85721 USA

**Keywords:** Gene regulatory networks, Network topology, Gene expression, Skin diseases, Diabetes complications, Type 2 diabetes, Biomarkers, RNA sequencing

## Abstract

Skin disorders are one of the most common complications of type II diabetes (T2DM). Long-term effects of high blood glucose leave individuals with T2DM more susceptible to cutaneous diseases, but its underlying molecular mechanisms are unclear. Network-based methods consider the complex interactions between genes which can complement the analysis of single genes in previous research. Here, we use network analysis and topological properties to systematically investigate dysregulated gene co-expression patterns in type II diabetic skin with skin samples from the Genotype-Tissue Expression database. Our final network consisted of 8812 genes from 73 subjects with T2DM and 147 non-T2DM subjects matched for age, sex, and race. Two gene modules significantly related to T2DM were functionally enriched in the pathway lipid metabolism, activated by *PPARA* and *SREBF* (*SREBP*). Transcription factors *KLF10*, *KLF4*, *SP1*, and *microRNA-21* were predicted to be important regulators of gene expression in these modules. Intramodular analysis and betweenness centrality identified *NCOA6* as the hub gene while *KHSRP* and *SIN3B* are key coordinators that influence molecular activities differently between T2DM and non-T2DM populations. We built a TF-miRNA-mRNA regulatory network to reveal the novel mechanism (*miR-21-PPARA-NCOA6*) of dysregulated keratinocyte proliferation, differentiation, and migration in diabetic skin, which may provide new insights into the susceptibility of skin disorders in T2DM patients. Hub genes and key coordinators may serve as therapeutic targets to improve diabetic skincare.

## Introduction

Diabetes mellitus is a long-term metabolic disease that has become a global public health threat over the past two decades. Approximately 463 million people are diagnosed with diabetes globally with an estimated increase of 50% in the next 25 years^1^. People with diabetes suffer from high blood glucose known as hyperglycemia which can affect every part of the human body resulting in a number of serious diseases. Patients with type 1 and type 2 diabetes often have skin complications that vary in occurrence from 51.1 to 97% in different regions worldwide^2^. Specifically, diabetic patients have a higher risk of cutaneous diseases and especially skin infections. Skin lesions result from poor healing in diabetic patients and can develop into chronic and recurrent ulcers causing difficulties in the clinical treatment of diabetes^2,3^.

Despite the fact that glucose dysregulation is well-known to impair the immune system and cellular skin function, the molecular mechanisms that are responsible for the deficiency of skin in diabetic patients are understudied. Alteration in gene expression is an indicator of biological activity that can help to identify genetic determinants of diseases. Recent studies with comparative high-throughput sequencing analysis of the skin in type 2 diabetes (T2DM) versus non-T2DM have noted a number of differentially expressed (DE) genes that are related to immune response, infectious disease, and structural changes in diabetic skin^4,5^. DE analyses consider the expression of individual genes between sample cohorts but ignore the mutual effect between genes in biological systems. Instead of working alone, genes interact with each other that jointly affect human health. A gene that is not differentially expressed across experimental conditions may still play an important role in linking biological networks that drive patterns of gene activity in pathological conditions.

The development of next-generation sequencing (NGS) technologies and transcriptomics (RNA-seq) allows for the quantification of gene expression in 1000’s genes simultaneously. Recently, RNA-seq data has been used in conjunction with integrative network analyses to identify and prioritize potentially disease-related genes. Beyond individual genetic alteration, the network-based analysis offers a systematic view of all genes by taking account of the interaction between each pair of genes. As genes with similar functions or that participate in the same biological pathway often exhibit coordinated expression patterns^6,7^, the gene co-expression network connected by quantitative co-expression similarity can be used for gene module identification and subsequent inference of gene functions and module-disease associations. In this study, we performed weighted gene co-expression network analysis (WGCNA) of the lower leg skin samples from the Genotype­Tissue Expression (GTEx) database to reveal the molecular mechanisms behind the susceptibility to skin disorders in T2DM patients. By analyzing gene co-expression patterns in skin cells between T2DM and non-T2DM subjects, we provide new insights into understanding the pathogenesis of T2DM skin disorders to advance diabetic skin care in clinics. In particular, we reveal a novel mechanism (*miR-21-PPARA-NCOA6*) of dysregulated keratinocyte proliferation, differentiation, and migration in diabetic skin. *NCOA6* is a coregulator involved in growth, development, energy homeostasis, and wound healing that may be a fundamental biomarker for managing skin care in diabetes^8^.

## Methods

### Data

The data of skin samples (the sun-exposed region of the lower legs) used in this research was obtained from the GTEx database (v7, September 2017 release). Detailed information on sample collection, RNA sequencing, and the data processing pipeline is described in the GTEx consortium paper^9^. The datasets involved in this research (summarized in Supplementary Table S1) are available from dbGaP (study accession number phs000424.v7.p2) and were carried out in accordance with relevant guidelines and regulations. We excluded cases with type 1 diabetes and/or unknown T2DM status from 751 participants, leaving 256 non-T2DM and 75 T2DM samples. To reduce the effects of confounding factors between non-T2DM and T2DM groups, we balanced the covariates of sex, age, and race using the R package “MatchIt” with the matching method “optimal” and matching ratio 2:1^10^. Following this process, 150 non-T2DM and 75 T2DM samples remained, with 56,202 RNA-seq transcriptome profiles.

### RNA-seq data preprocessing

Since low-expressed or non-varying genes usually indicate noise, we filtered out the intersection of the genes with read counts of less than 10 in more than 90% of samples in each group. We then retained the top 50% most variable genes based on the MAD of gene expression values (represented by TPM values described in Supplementary Table S1) across all the samples. A total of 8812 out of 56,202 genes were selected, and their TPM values were then log-transformed using $\log_{2} \left( {x + 1} \right)$. As GTEx data are highly heterogeneous and affected by batch effects, it is common practice to adjust known and hidden confounders in GTEx gene expression data^11,12^. Based on the known adjustment variables (gender, age, BMI, and ischemic time) and the variable of interest (T2DM status), we applied the R package “sva” to estimate surrogate variables (SVs) for latent sources of noise^13^ and picked 3 most significant SVs for correction on TPM expression matrix.

Though DE genes in T2DM skin have been reported by previous studies, it is not convincing to assess the findings from current network analysis versus previous DE analyses due to the differences in the datasets and preprocessing methods. Therefore, we also performed a DE analysis with DESeq2^14^ on this same cohort following similar preprocessing steps described above. As the DESeq2 internally corrects for the library size, the input should be the raw count data prior to TPM normalization. The estimated SVs were added to the “DESeqDataSet” design formula and DE genes were identified with adjusted *p* value $\le 0.05$.

### Sample outliers detection

Due to variability in the data acquisition, outliers may occur that can influence the robustness of the downstream analysis. Sample networks have been demonstrated to be an appropriate method for outlier detection because it is independent of the choice of clustering algorithms and can clearly distinguish samples, particularly among large genomic data^15^. We identified 3 outliers in the non-T2DM group and 2 outliers in the T2DM group respectively through a Euclidean distance-based sample network, with standardized connectivity lower than 2.5 ($Z \cdot k < - 2.5$) (Supplementary Fig. S1).

### Weighted co-expression network construction

In gene co-expression networks, nodes correspond to genes, and edges represent the magnitude of their co-expression. Unweighted networks may lead to a loss of information when determining the connectivity between genes using binary parameters (1 or 0). Because most biological networks are scale-free networks, their degree distribution follows a power law $p\left( k \right)\sim k^{ - \gamma }$. Based on this property, Zhang and Horvath 2005 have proposed a scale-free topological criterion to assign connection weight to each gene pair in weighted gene co-expression network analysis (WGCNA)^16^.

The R package “WGCNA”^17^ was used to perform network construction. Briefly, the absolute value of the Pearson correlation $s_{ij} = \left| {cor\left( {i,j} \right)} \right|$ was calculated, which measures the similarity between the expression profiles of genes $i$ and $j$. Then, the co-expression similarity $s_{ij}$ was transformed into $a_{ij}$ via power adjacency function $a_{ij} = s_{ij}^{\beta }$. In this function, $\beta \ge 1$ is a soft threshold determined by the scale-free topological criterion and is known as the model fitting index $R^{2}$ of the linear model that regresses $\log \left( {p\left( k \right)} \right)$ on $\log \left( k \right)$. After the soft thresholding parameter $\beta$ was determined, the topological overlap matrix ($TOM$)^16,18^ was obtained to describe the weight of the edge between genes.

### Gene module identification

A gene module is a subset of nodes (or genes) that are highly connected. WGCNA uses a topological overlap dissimilarity measure ($dissTOM = 1 - TOM$)^16^ to identify these modules, which has been shown to result in biological meaningful modules when clustered using hierarchical clustering. Inside each module, the first principal component is defined as the module eigengene (ME) representing its overall expression level.

### Module-trait associations and intramodular analysis

The module-trait association is defined by the *p* value of the correlation between the expression level of each ME and each trait variable $cor\left( {ME, trait} \right)$. The trait of interest is the T2DM status, where 0 indicates non-T2DM, and 1 indicates T2DM. The gene significance (GS) of each clinical trait is defined as the absolute value of the correlation between *i*th gene expression profile $x_{i}$ and the sample trait $cor\left( {x_{i} , trait} \right)$. Genes with high absolute values of GS represent their individual significant associations with the phenotypic trait. The module membership (MM) is defined as the correlation between the expression level of ME and the *i*th gene $cor\left( {ME, x_{i} } \right)$ that represents how close a gene is to a given module. Higher absolute values of MM indicate greater similarity between the genes and module eigengene. Generally, intramodular hub genes exhibit high MM values. The hub genes from the modules significantly associated with T2DM were regarded as potential driver genes.

### Identification of hub genes with betweenness centrality

Intramodular hubs are central to specific modules in the network and are frequently more relevant to the functionality than other nodes^19^. Betweenness centrality (BC) is often used to identify hub genes as it is a network topological characteristic that quantifies the ability of a node that transfers information in a module. Nodes with high BC are considered to be more biologically informative that are responsible for transferring communication information. Comparison of such property between two networks can reveal the alterations in co-expression patterns of a particular gene and its connected portions under two states. Therefore, we constructed T2DM and non-T2DM gene co-expression networks separately based on their respective $TOM$ and performed centrality analysis of the genes from T2DM-related modules via R package Igraph^20^. The inverse of topological overlap represents the length of an edge and BC is defined as the number of shortest paths between every two other nodes in the module that pass through that node (Eq. (1)).

1 $$BC\left( v \right) = \mathop \sum \limits_{i \ne j, i \ne v, j \ne v } \frac{{g_{ivj} }}{{g_{ij} }}$$

where $v$ is the set of nodes, $g_{ij}$ is the number of shortest paths between nodes $i$ and $j$, and $g_{ivj}$ is the number of those paths that pass through node $v$. The genes with considerable changes in BC values between T2DM and non-T2DM networks might be the driver genes influencing health status.

### Enrichment analysis and visualization

To understand the underlying biological mechanisms indicated in T2DM-related modules, enrichment analyses were performed for gene ontologies (GO), KEGG pathways, and Reactome pathways with DAVID^21^ and g:Profiler^22^. Significant gene sets were restricted to the ones with size $\ge 5$ at an adjusted *p* value of 0.05, then visualized with EnrichmentMap^23^ in Cytoscape^24^. In addition to the gene itself, the regulatory motifs also play essential roles upstream of gene expression. TFs help initiate gene transcription by binding promoter regions of the target DNA at the transcriptional level while miRNAs regulate protein translation by binding the 3’ untranslated region of the target mRNA at the post-transcriptional level. g:Profiler was employed to predict the TFs and miRNAs involved in the regulation of disease genes based on the databases of TRANSFAC and miRTarBase^22^. The resulting TFs, miRNAs, and their target genes were used to build a regulatory network visualized via Cytoscape^24^.

## Results

### Co-expression network generation with WGCNA

**Figure 1:**
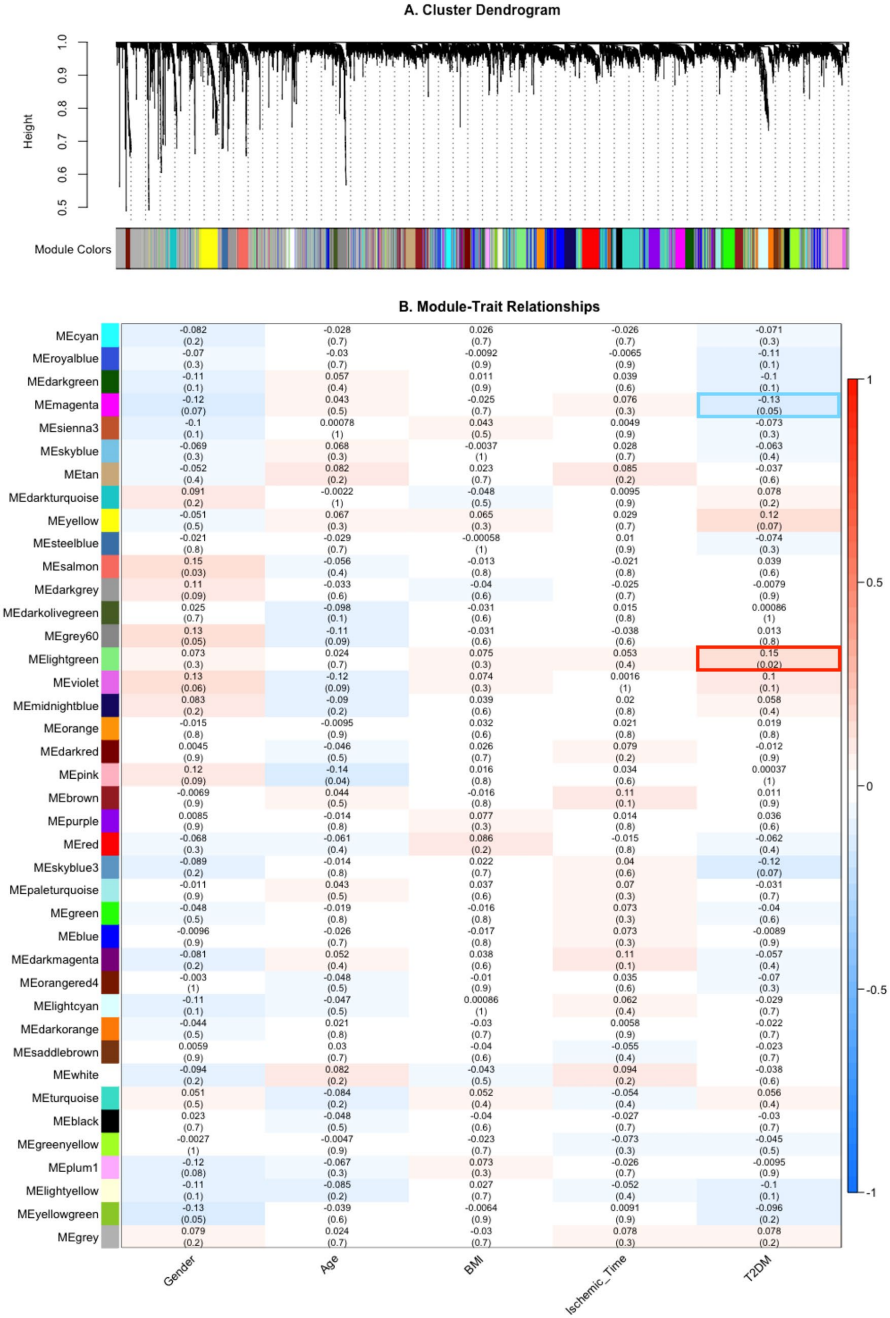
Gene co-expression network generation. (**A**) Cluster dendrogram denoting gene modules: based on hierarchical clustering of topological overlap dissimilarity, modules were identified and denoted with different colors. (**B**) Module-trait relationships: the heatmap represents is a correlation matrix representing the correlation and *p* value (in parentheses) between each ME and each trait.

Skin samples from the GTEx database were classified into T2DM and non-T2DM groups. Based on data preprocessing and sample outlier detection, 8812 most varied genes from 73 subjects with T2DM and 147 non-T2DM subjects matched for age, sex, and race were imported to WGCNA to detect gene modules significantly correlated with T2DM. $\beta = 7$ was chosen for the power of adjacency function as it is the point ($R^{2} = 0.86$) at which the scale-free topology model fit tends to level off (Supplementary Fig. S2). Based on hierarchical clustering of topological overlap dissimilarity, 39 modules were identified with module sizes ranging from 757 to 55 genes and denoted with different colors where the grey module contained all unassigned genes (Fig. 1A). The module-trait relationships measured by the *p* value of $cor\left( {ME, trait} \right)$ showed that 2 modules colored in lightgreen (*p* value $= 0.02$) and magenta (*p* value $= 0.05$) had significant associations with T2DM status (Fig. 1B). As for DE analysis, 13 out of 8812 genes were identified as DE genes (Supplementary Table S2) and there was no overlap between DE genes and the genes from the T2DM-related modules.

### Intramodular analysis of significant modules

**Figure 2:**
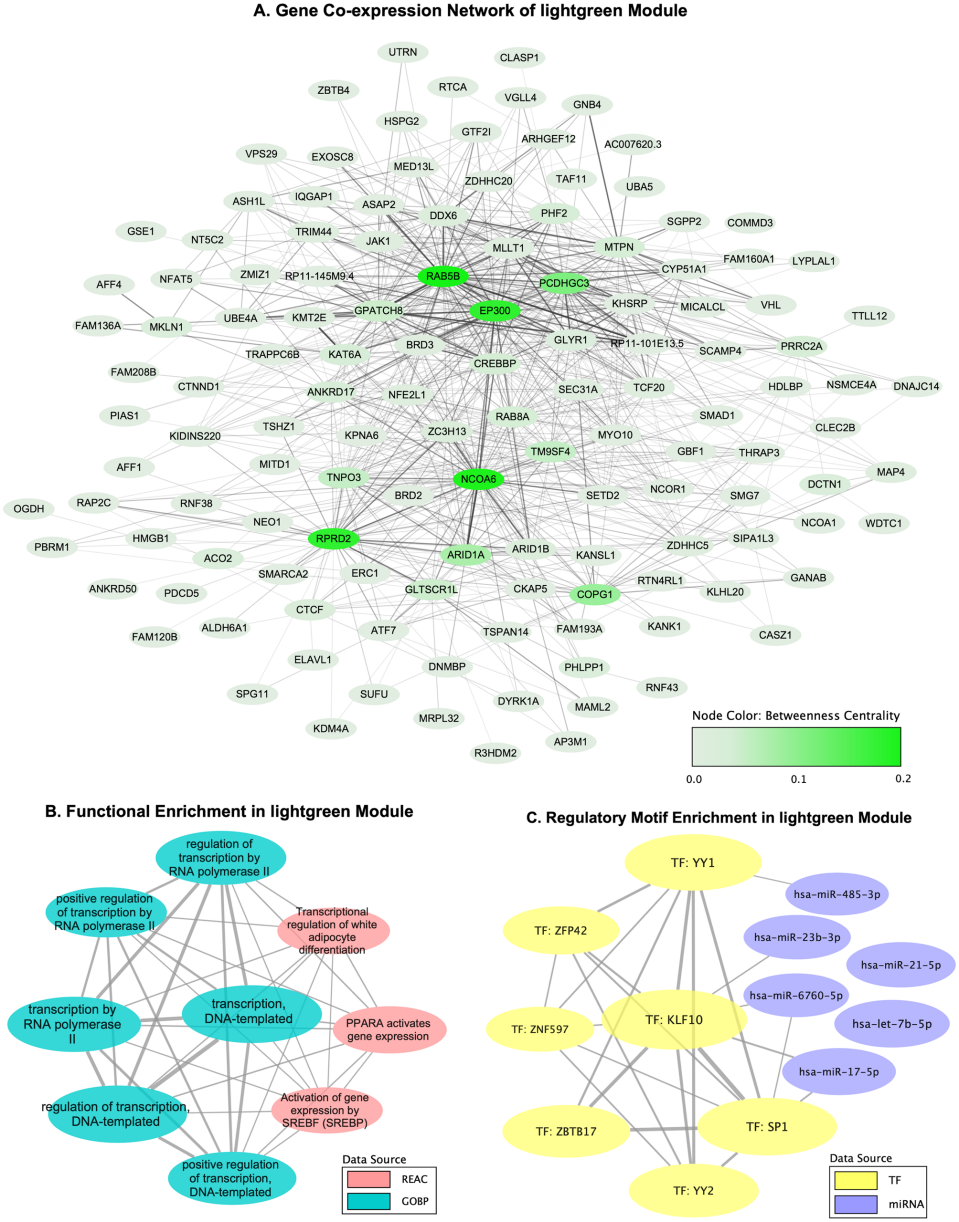
Intramodular analysis of lightgreen module. (**A**) Gene co-expression network of lightgreen module. (**B**) Functional enrichment of lightgreen module: each node is a gene set and the width of an edge represents the number of genes shared by every two gene sets. (**C**) Regulatory Motif enrichment of lightgreen module: each node is a gene set representing predicted targets of regulation by a TF or miRNA and the width of an edge represents the number of target genes shared by every two TF/miRNA.

**Figure 3:**
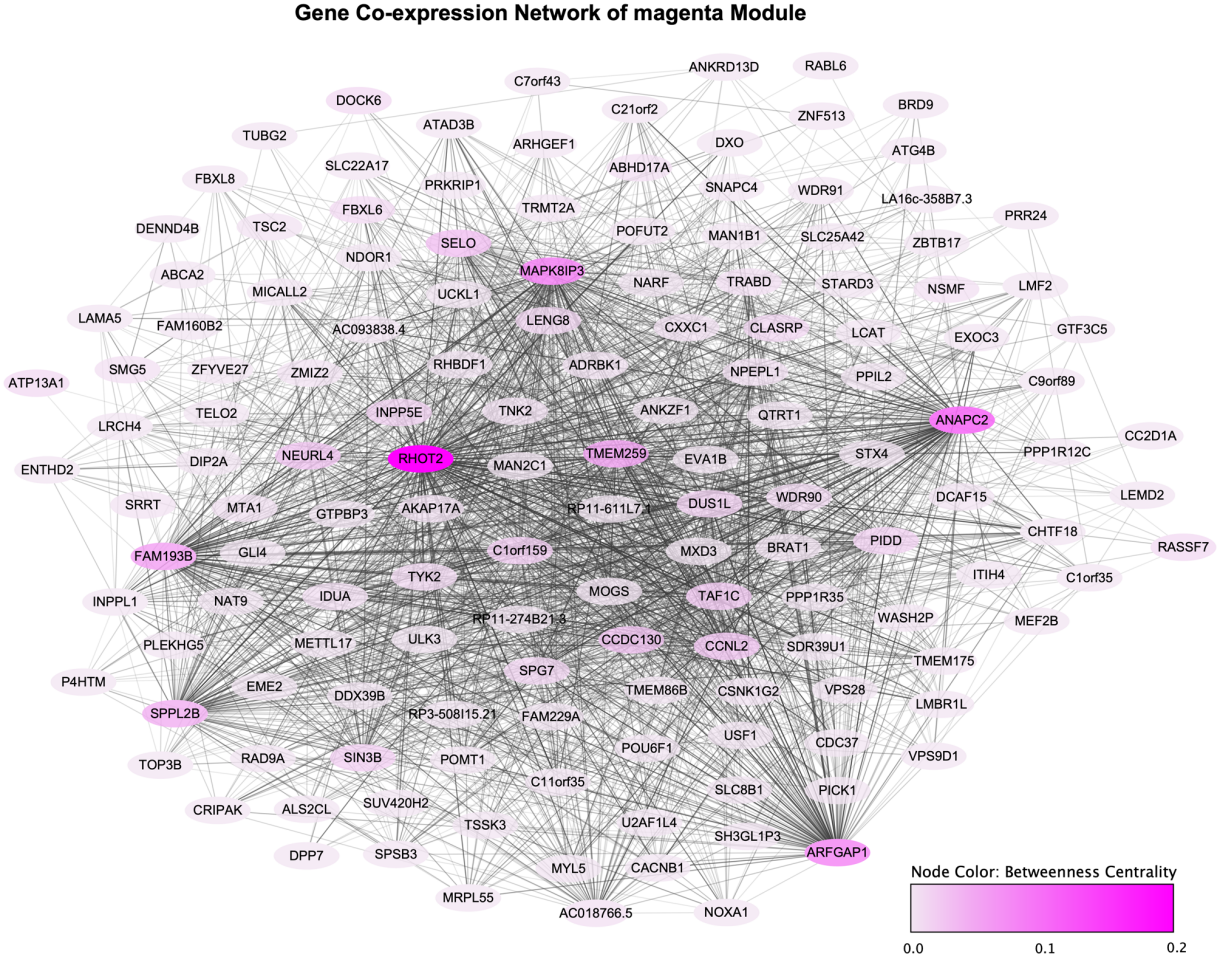
Gene co-expression network of magenta module.

The lightgreen module included 145 genes while the magenta module included 213 genes. To identify the key genes in these two T2DM-related modules, we used module membership (MM), gene significance (GS), and betweenness centrality (BC) to measure their intramodular properties. Table 1 provides the top 10 genes from each module by the absolute values of their MM, GS, and BC. The entire gene list can be found in Supplementary Tables S3 (lightgreen module) and S4 (magenta module). The top-ranked genes based on the value of MM were considered as the representatives of a module, such as *NCOA6*, *RAB5B*, *EP300*, and *CREBBP* in the lightgreen module, and *RHOT2* in the magenta module. The GS in both modules was highly correlated with MM (Supplementary Fig. S3), indicating that these intramodular representatives were often significantly associated with T2DM status. The topological analysis with betweenness centrality also showed that *NCOA6*, *EP300*, and *RAB5B* in the lightgreen module and *RHOT2* in the magenta module had the highest BC, which further confirmed that they were the intramodular hubs that carried more biologically relevant information and played the role of an important connector throughout a module (Figs. 2A and 3).

**Table 1:** Top 10 genes in lightgreen and magenta modules.

Gene ID	Description	MM.lightgreen	GS.T2DM	p.GS.T2DM	BC.lightgreen
**Top 10 genes in lightgreen module**					
NCOA6	Nuclear receptor coactivator 6	0.881	0.181	0.007	2150
EP300	E1A binding protein p300	0.859	0.142	0.035	2008
RAB5B	RAB5B, member RAS oncogene family	0.857	0.131	0.052	1977
RPRD2	Regulation of nuclear pre-mRNA domain containing 2	0.855	0.134	0.046	1361
TNPO3	Transportin 3	0.848	0.138	0.041	216
ARID1A	AT-rich interaction domain 1A	0.836	0.148	0.028	265
TM9SF4	Transmembrane 9 superfamily member 4	0.825	0.112	0.099	232
CREBBP	CREB binding protein	0.811	0.095	0.162	57
ZC3H13	Zinc finger CCCH-type containing 13	0.81	0.163	0.016	22
RAB8A	RAB8A, member RAS oncogene family	0.81	0.097	0.152	89
**Top 10 genes in magenta module**					
RHOT2	RAS homolog family member T2	0.919	− 0.168	0.012	12,299
ARFGAP1	ADP ribosylation factor GTPase activating protein 1	0.889	− 0.189	0.005	1362
ANAPC2	Anaphase promoting complex subunit 2	0.884	− 0.148	0.028	4523
MAPK8IP3	Mitogen-activated protein kinase 8 interacting protein 3	0.875	− 0.121	0.073	1165
TMEM259	Transmembrane protein 259	0.864	− 0.141	0.037	643
TAF1C	TATA-box binding protein associated factor, RNA polymerase I subunit C	0.86	− 0.113	0.094	13
FAM193B	Family with sequence similarity 193 member B	0.856	− 0.121	0.074	672
SPPL2B	Signal peptide peptidase like 2B	0.854	− 0.08	0.238	791
C1orf159	Chromosome 1 open reading frame 159	0.843	− 0.133	0.049	0
SELO	Protein Adenylyltransferase SelO	0.83	− 0.186	0.006	19

Furthermore, T2DM and non-T2DM networks were generated separately to compare the sub-networks of T2DM-related modules between T2DM and nonT2DM population (Supplementary Figs. S4 and S5). The genes with dramatic changes in BC values between T2DM and non-T2DM networks were regarded as coordinator candidates that might jointly influence molecular activities with hubs. Beyond the hub genes mentioned above, other genes with a large difference in BC value were identified like *CREBBP* and *KHSRP* in the lightgreen module and *SIN3B* in the magenta module (Supplementary Tables S5 and S6). For example, *KHSRP* had high BC ($BC_{T2DM} = 612$) in the T2DM network but low BC ($BC_{non - T2DM} = 0$) in the non-T2DM network, suggesting that it played a different role in information transfer between two networks. The features MM, GS, and BC can be combined with the following functional analysis to help determine the biological importance of identified hub genes and candidate gene markers.

### Functional analysis and pathway enrichment

To explore the biological meaning and underlying mechanisms in T2DM-related modules, enrichment analysis of functional pathways and prediction of regulatory motifs were performed with DAVID and g:Profiler. GO analysis revealed that genes in the lightgreen module were involved in 6 biological processes (BP) relevant to transcription, including the cellular synthesis of RNA on a DNA template and the synthesis of RNA mediated by RNA polymerase II. Meanwhile, 3 Reactome pathways were enriched under the metabolism of lipids, including *PPARA* activating gene expression, transcriptional regulation of white adipocyte differentiation, and activation of gene expression by *SREBF* (*SREBP*) (Fig. 2B). Intramodular hub gene *NCOA6* was shown to participate in all BPs and pathways and gene *EP300* and *CREBBP* also played important roles in most of these processes (Supplementary Table S7). Enrichment of regulatory motifs predicted 13 gene sets, including interested genes of *NCOA6*, *EP300*, and *CREBBP*, as the targets of regulation by 7 TFs *KLF10*, *SP1*, *YY1*, *YY2*, *ZNF597*, *ZBTB17*, and *ZFP42* and 6 miRNAs *hsa-let-7b-5p*, *hsa-miR-6760-5p*, *hsa-miR-23b-3p*, *hsa-miR-21-5p*, *hsa-miR-485-3p*, and *hsa-miR-17-5p* (Supplementary Table S7, Fig. 2C).

Interestingly, no GO BPs or functional pathways were identified, however 410 TF motifs were significantly enriched from the TRANSFAC database that regulated the genes in the magenta module (Supplementary Table S8). The motifs mainly belong to SP/KLF, E2F, YY1/YY2, and AP-2 families. Key genes such as RHOT2, INPP5E, and SIN3B were all predicted as the targets of these TFs.

### Regulatory network of TF-miRNA-mRNA

**Figure 4:**
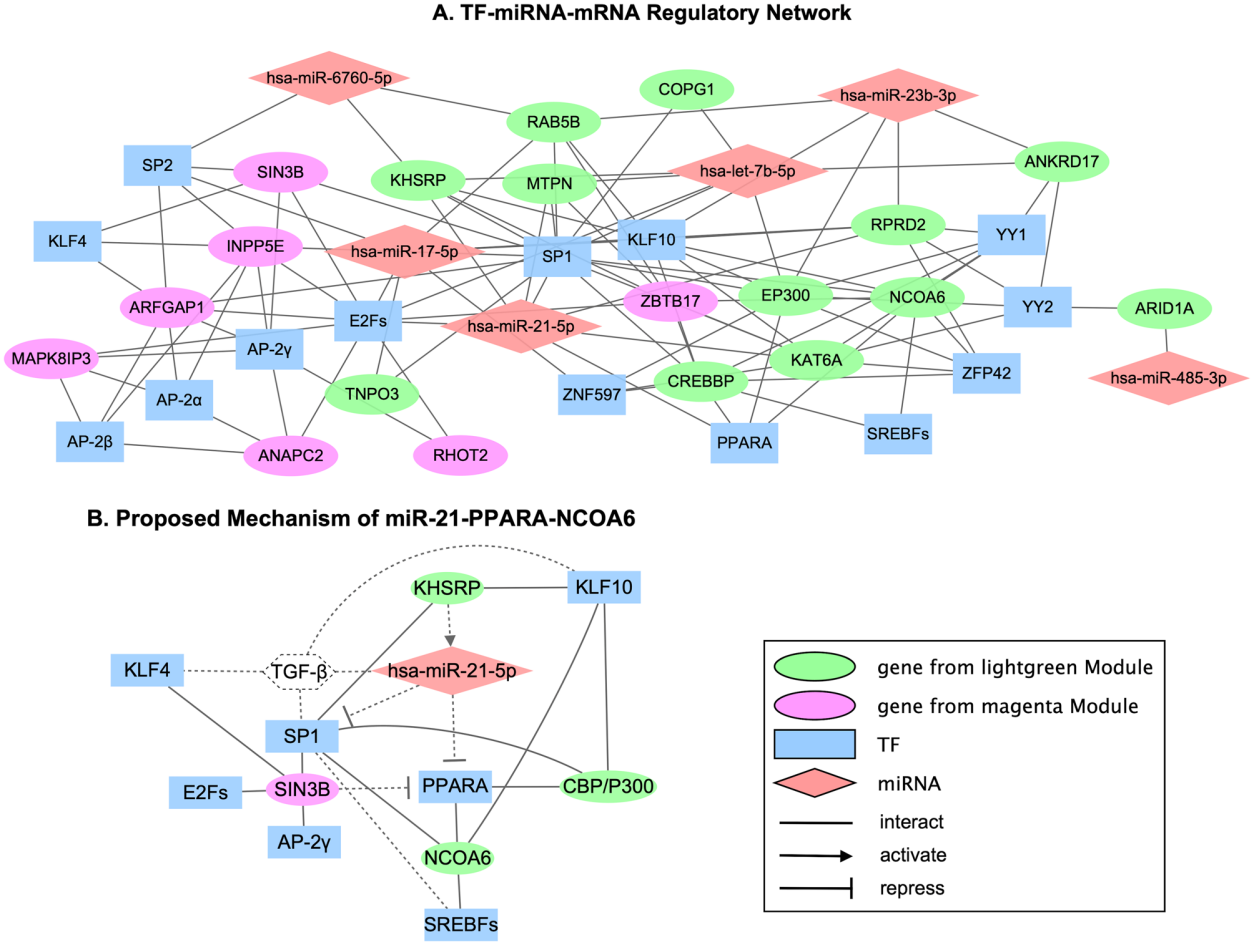
Regulatory network of T2DM-related modules. (**A**) TF-miRNA-mRNA network of top genes from lightgreen and magenta modules. (**B**) Proposed mechanism of *miR-21-PPARA-NCOA6*: a simplified TF-miRNA-mRNA network for hub genes and key regulators, which is associated with keratinocyte proliferation, differentiation, and migration. The solid line represents the interaction obtained from enrichment analysis while the dash line represents the potential interaction based on the previous publications.

Following an enrichment analysis of the genes in the lightgreen and magenta modules for potential TFs and miRNAs in the TRANSFAC and miRTarBase databases separately, a TF-miRNA-mRNA interaction network was constructed and visualized by Cytoscape (Fig. 4A). The TF-miRNA-mRNA interaction network consisted of 19 key genes that were selected based on MM, GS, and BC, along with 14 TFs and 6 miRNAs. The gene *ZBTB17* from the magenta module was also the TF enriched in the lightgreen module, which regulated the expression of hub genes (*NCOA6*, *RAB5B*) and other important candidates (*CREBBP*, *KHSRP*). Besides, the TF *PPARA* that activated the expression of lightgreen genes (*NCOA6*, *EP300*, *CREBBP*) were shown to be the target repressed by magenta gene *SIN3B*. In particular, we found a sub-regulatory network that centered on *miR-21* which was one of the most well-proven miRNAs associated with inflammation and keratinocyte proliferation in the skin during wound healing (Fig. 4B). Hub gene *NCOA6*, key coordinators (*KHSRP* and *SIN3B*), and major TFs (*PPARA*, *SREBFs*, *KLF10*, *KLF4*, *SP1*, etc.) were all involved in this sub-network.

## Discussion

Hyperglycemia is a hallmark characteristic of Type II diabetes that affects every part of the body including the skin. Diabetes-associated skin problems have become one of the most common complications of diabetes. In particular, chronic ulcers, or severe cutaneous lesions, occur due to impaired wound healing in diabetic skin. The high prevalence of skin disorders among people with diabetes makes it essential to understand molecular mechanisms underlying changes in the skin. Our network-based analysis provides insights into connections between genes, where the aberrant coordinated expression patterns across a variety of experimental conditions can reflect disease-related biological processes. Therefore, the present study explores the biological pathways, disease driver genes, and TF-miRNA-mRNA regulatory programs in human skin that are associated with T2DM using WGCNA in combination with other network topology characteristics.

According to the results from WGCNA, two modules that were significantly associated with T2DM were chosen for functional enrichment analysis. The genes in the lightgreen module were involved in the GO biological process of transcription which mainly belonged to the terms of synthesis of RNA on a DNA template mediated by RNA polymerase II. The enriched Reactome pathways showed that the lightgreen module participated in (1) the regulation of lipid metabolism by *PPARA* and *SREBFs*; (2) transcriptional regulation of white adipocyte differentiation. Lipids play an important role in the formation and maintenance of skin barriers that support the movement of water and electrolytes and protect against invasive microbes. Meanwhile, the layer of adipocytes within the reticular demis known as dermal white adipose tissue also provides essential functions that contribute to the skin barrier integrity, such as antimicrobial defense and wound healing^25^. The *PPARA* and *SREBF* genes that arose in the enrichment results are both transcription factors that act as key regulators of lipid metabolism to maintain skin homeostasis. *PPARA* (also referred to as *PPAR-α* or *NR1C1*) is a member of the peroxisome proliferator-activated receptors that have been found to control keratinocyte proliferation/differentiation, regulate skin inflammation, and contribute to skin repair after injury^26^. For example, *PPARA*-deficient mice show a delay in wound healing compared to wild-type mice, especially during the initial inflammatory phase^27^. *SREBFs* (also referred to as *SREBPs*) are sterol regulatory element-binding proteins responsible for cholesterol biosynthesis. Beyond the importance of *SREBFs* in epidermal differentiation and skin barrier formation^28^, studies have highlighted that *SREBF2* limits wound healing via tumor necrosis factor (TNF)-induced mechanisms^29^.

The lightgreen intramodular hub gene *NCOA6*, along with gene *EP300* and *CREBBP*, were the most common genes shared by the above pathways. Studies with gene knockout mice have demonstrated that *NCOA6* (nuclear receptor coactivator 6), *EP300* (also known as *P300* or *E1A* binding protein *P300*), and *CREBBP* (also known as *CBP* or *CREB*-binding protein) are essential coactivators in systemic biology and physiology^8^. *NCOA6* and *CBP/P300* can interact with each other, and both have the ability to enhance the activity of a wide variety of other transcription factors involved in growth, proliferation, cytokine signaling, metabolism, immune response, and apoptosis^8^. Particularly, *NCOA6* is a *PPAR*-interacting protein that can function as a coactivator to regulate *PPARA*. In addition, *NCOA6*^+/−^ mice have been shown to exhibit a wound-healing phenotype where a defect in keratinocyte migration found in the skin of *NCOA6*^+/−^ adult mice leds to chronic skin wounds when compared to wild-type^8,30^. The altered co-expression patterns of the lightgreen module centered on *NCOA6* may affect *PPARA* and *SREBFs* signaling pathways, which contributes to the susceptibility of the T2DM population to develop skin complications.

Moreover, TFs and miRNAs are now recognized as critical regulators that control the level of expression of target genes. Here, the TFs and miRNAs of T2DM-related modules were predicted separately with TRANSFAC and miRTarBase databases. TFs mainly belonged to *SP/KLF*, *E2F*, *YY1/YY2*, and *AP-2* families while miRNAs were *hsa-let-7b-5p*, *hsa-miR-6760-5p*, *hsa-miR-23b-3p*, *hsa-miR-21-5p*, *hsa-miR-485-3p*, and *hsa-miR-17-5p*. Subsequently, a co-regulation network was constructed, combined with the relationships reported in previous studies, to illustrate the interactions of TF, miRNA, and target genes (Fig. 4A). We primarily focused on the connections with *miR-21* (Fig. 4B) as it is highly expressed in the skin that regulates cell proliferation and migration, which has been found to be implicated in a variety of skin disorders such as psoriasis, scleroderma, dermatomyositis, and cutaneous melanoma^31,32^. In particular, *miR-21* plays multiple roles in the proliferation phase of wound healing. It promotes *TGF-β* (transforming growth factor-β)-driven keratinocyte migration in re-epithelialization^33^ but inhibits endothelial cells proliferation, migration, and tube formation during the angiogenesis process^34^. Studies have shown that the expression of *miR-21* in diabetic skin increased in response to the high level of glucose but decreased in diabetic wounds, which was opposite to the situation in normal skin^35^. Such aberrant regulation of *miR-21* induced by diabetes could be critical to the abnormal healing of chronic wounds. Although *miR-21* does not directly control the expression of hub gene *NCOA6*, it may regulate *NCOA6* by suppressing *PPARA*^36^. Meanwhile, topological analysis with betweenness centrality revealed that the lightgreen gene *KHSRP* (KH-type splicing regulatory protein), which had been found to promote the maturation of *miR-21*^37^, acted as an important node in information transfer in the T2DM network ($BC_{T2DM} = 612$) but not in the non-T2DM network ($BC_{non - T2DM} = 0$). The obvious difference of *KHSRP* between the two networks may affect the *miR-21* biogenesis and relevant *PPARA*-mediated pathways which further contribute to diabetic skin disorders. Another gene with a large difference in BC value between the non-T2DM and T2DM networks was *SIN3B* (*SIN3* transcription regulator family member B) from the magenta module, which was a corepressor of *PPARA* under the pathway of lipid metabolism. *SIN3B* exhibited a very high BC in the T2DM network ($BC_{T2DM} = 3976$) but low BC in the non-T2DM network ($BC_{non - T2DM} = 5$), which might indicate the inhibition of *PPARA*-mediated pathways in the T2DM population.

Additionally, enrichment analysis identified that *KLF4* (Kruppel-like factor 4), known to be required for establishing the barrier function of the skin^38^, was one of the most significant transcription factors targeting magenta genes including SIN3B. Studies have shown the importance of *KLF4* in keratinocyte precursor immaturity^39^ and have found that it can facilitate cutaneous wound healing by promoting the generation of fibrocytes, which serve as effector cells enhancing keratinocyte proliferation^40^. Other SP/KLF factors like *KLF10* and *SP1* were also predicted as main TFs that regulated disease modules including hub gene *NCOA6* and gene pair *CBP/P300*. *KLF10*, originally named *TGF-β* inducible early gene 1, plays a role in *TGF-β*-induced collagen synthesis and reepithelialization^41^ while *SP1* is involved in *TGF-β*-induced collagen synthesis in skin fibroblasts as well^42^. The other two broadly enriched TF families, *E2F* and *AP-2*, were also considered key regulators of modules associated with T2DM. *E2F* is known as a regulator of keratinocyte proliferation, of which *E2F1*, *E2F2*, and *E2F3* are the top enriched factors in our analysis that have been found relevant to the maintenance of the proliferation phase in primary keratinocytes during wound repair^43^. Similarly, the enriched *AP-2* members (*AP-2α*, *AP-2β*, *AP-2γ*) also have been shown to be involved in epidermal differentiation and barrier formation^44^.

The above results consistently suggest that the T2DM-related modules contain functions that are important for skin development. Important genes identified by network analysis and topological properties in the current study like *NCOA6*, *KHSRP*, and *SIN3B* have not been reported as DE genes in the previous work^4,5^ as well as in our DE analysis, which demonstrates that non-DE genes may also drive biological differences between healthy and disease states. The role of other genes included in these modules, such as the magenta hub gene *RHOT2*, is still unknown and remains to be further investigated. In addition, several limitations exist in this study. First, the current results are based on the lower leg skin samples from the GTEx database. With more applicable skin datasets released, a meta-analysis across different cohorts should be conducted in the future to identify generalizable mechanisms relevant to diabetic skin and verify the novel findings. Further experimental validation is needed to confirm the candidate biomarkers and their putative miRNAs and TFs. It is also of clinical importance to explore the signaling pathways involved in the mechanistic regulatory network for a better understanding of skin disorders in diabetic patients.

## Conclusions

In conclusion, we built a TF-miRNA-mRNA regulatory network with an emphasis on the regulations of hub gene *NCOA6* mediated by *PPARA* (*miR-21-PPARA-NCOA6*) to reveal a potential mechanism of dysregulated keratinocyte proliferation, differentiation, and migration in diabetic skin based on the enrichment analysis of T2DM-related gene modules detected by WGCNA. Gene *KHSRP* and *SIN3B* identified by betweenness centrality are considered important regulators of this proposed mechanism via interacting with *miR-21* and *PPARA* separately. Network-based analysis and topological properties allow us to expand insight into the pathogenesis of T2DM patients to develop skin disorders, which highlights the importance of the system biology approach to study complex diseases. The hub genes and key coordinators may be used as novel therapeutic targets to advance diabetic skin care.

## Supplementary Information

Supplementary Figures.

Supplementary Tables.

Supplementary Legends.

## Data Availability

All code for computational analysis is available at https://github.com/hurwitzlab/WGCNA-of-Type-II-Diabetic-Skin. The GTEx datasets can be downloaded from dbGaP with study accession number phs000424.v7.p2.
