# Supplementary material for: Network analysis reveals dysregulated functional patterns in type II diabetic skin
Source: Sci Rep. 2022 Apr 27;12:6889. doi: 10.1038/s41598-022-10652-8 (PMC9046425; doi:10.1038/s41598-022-10652-8)
Supplement: Supplementary file 1 — Supplementary Figures. [file 41598_2022_10652_MOESM1_ESM.pdf]

Network Analysis Reveals Dysregulated Functional Patterns in Type II Diabetic Skin

Chunan Liu[1], Sudha Ram[2], and Bonnie L. Hurwitz[1]*
[1] BIO5 Institute and Department of Biosystems Engineering, University of Arizona, Tucson, AZ, 85721, USA
[2] BIO5 Institute and Department of Management Information Systems, University of Arizona, Tucson, AZ, 85721, USA
* To whom correspondence should be addressed. Phone: 520-626-9819; Email: bhurwitz@email.arizona.edu

Supplementary Figures S1-S5

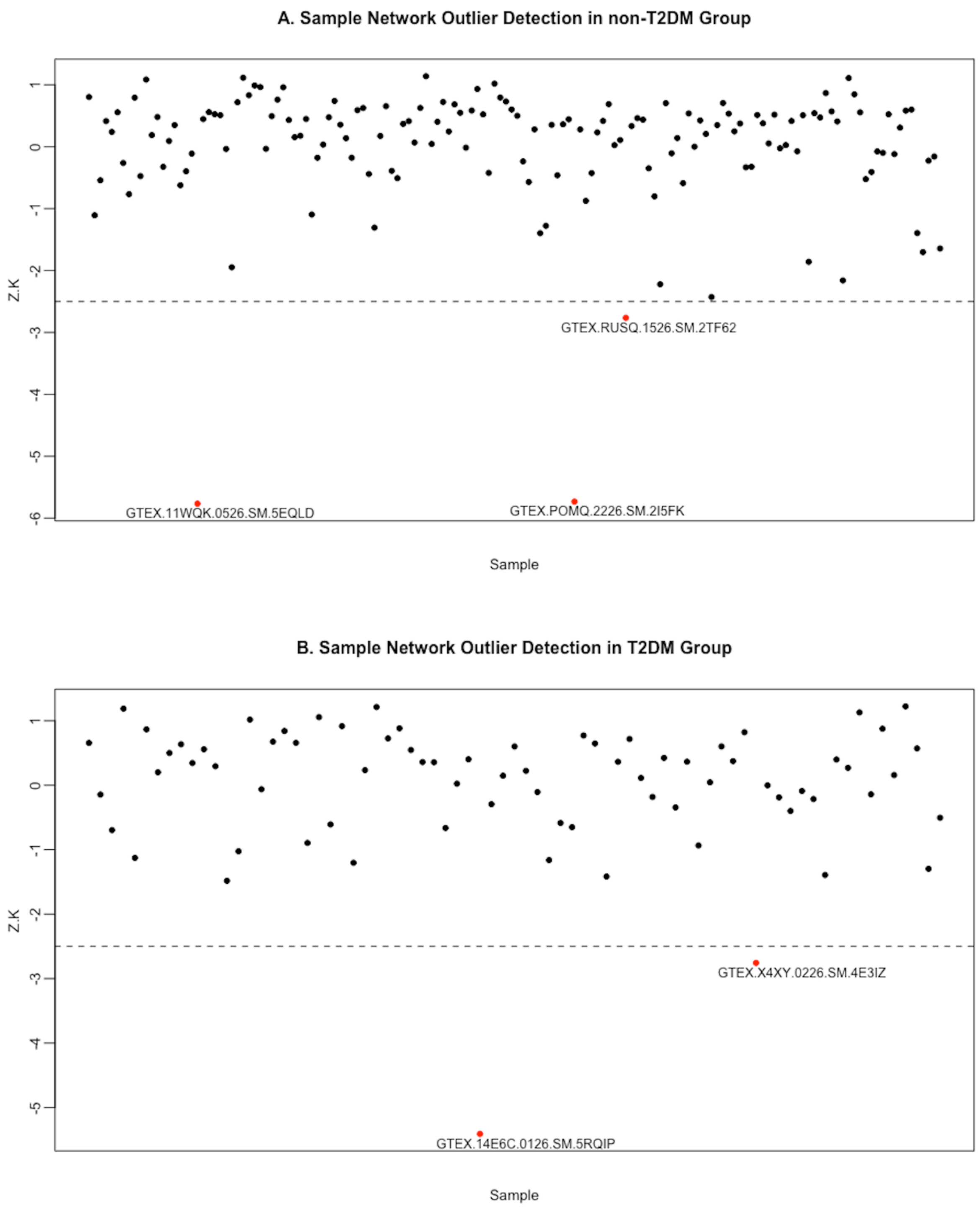

Supplementary Figure S1. Sample Outlier Detection

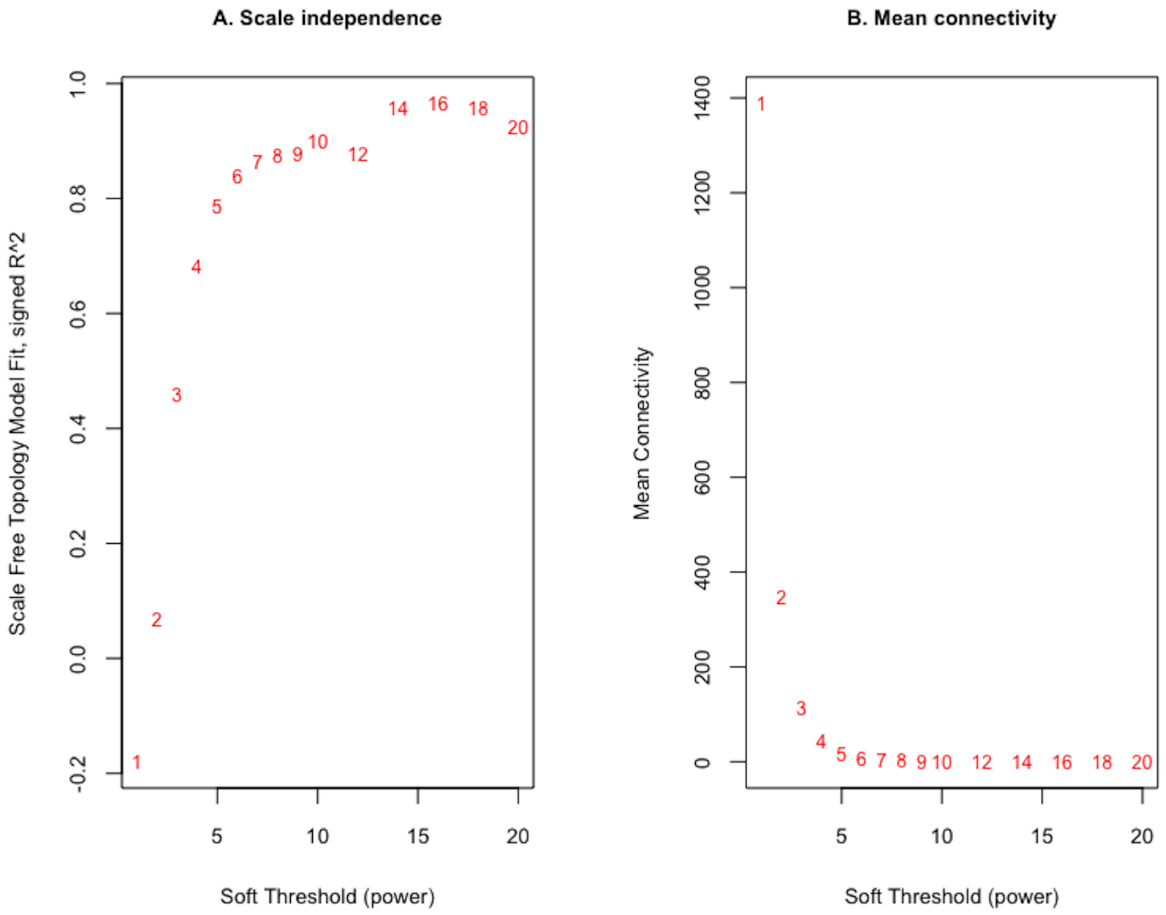

Supplementary Figure S2. Scale-free Topological Criterion

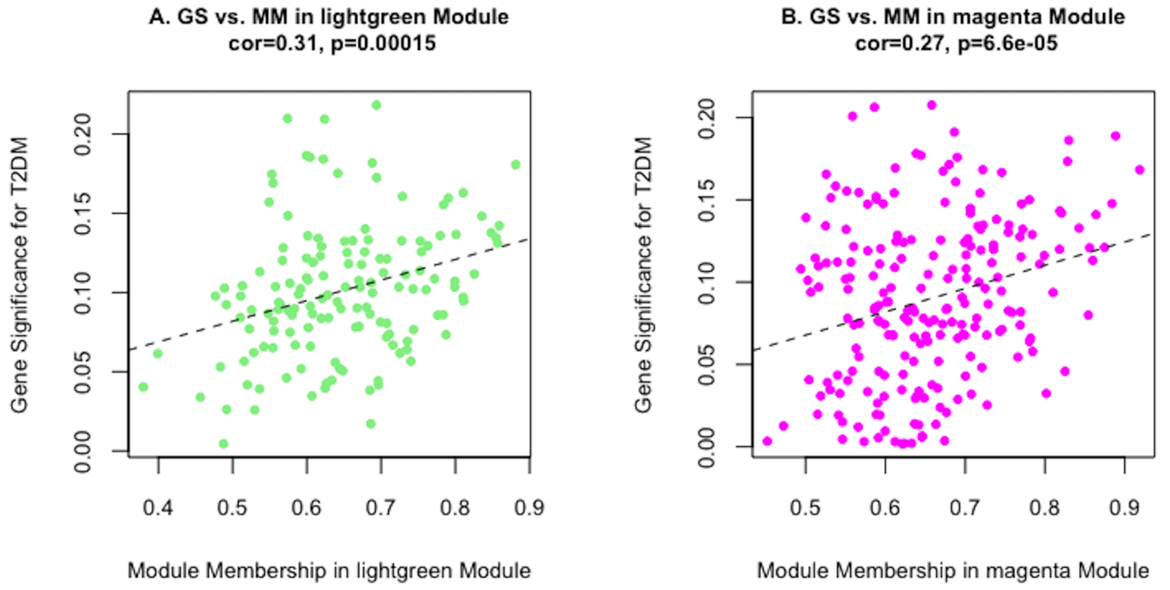

Supplementary Figure S3: (A) GS vs. MM for lightgreen module. (B) GS vs. MM for magenta module.

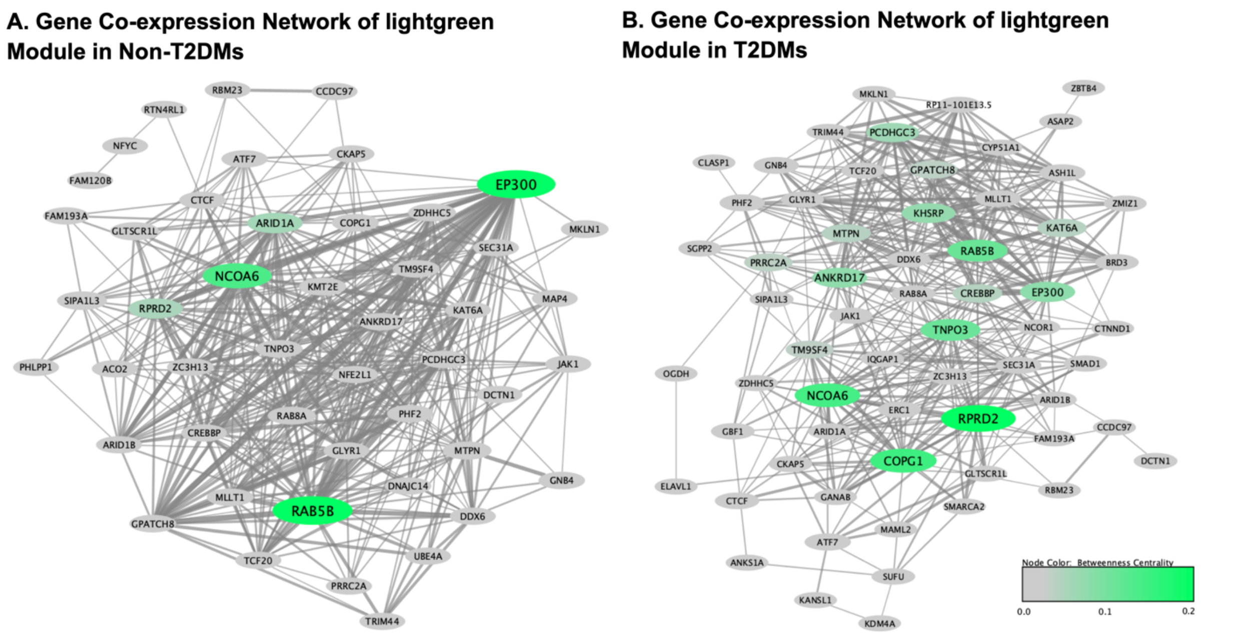

Supplementary Figure S4: (A) Sub-network of lightgreen module in non-T2DMs: 48 genes from lightgreen module with $BC > 0$ were extracted from non-T2DM networks for sub-network generation. (B) Sub-network of lightgreen module in T2DMs: 63 genes from lightgreen module with $BC > 0$ were extracted from T2DM networks for sub-network generation.

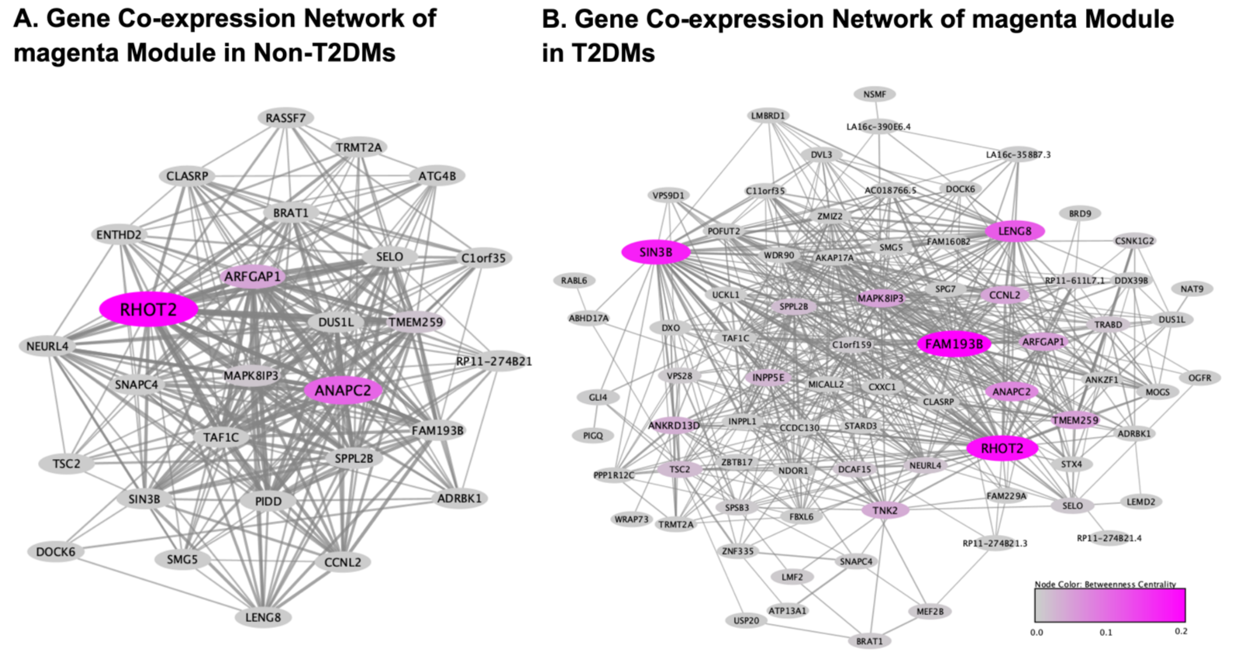

Supplementary Figure S5: (A) Sub-network of magenta module in non-T2DMs: 28 genes from magenta module with $BC > 0$ were extracted from non-T2DM networks for sub-network generation. (B) Sub-network of magenta module in T2DMs: 78 genes from magenta module with $BC > 0$ were extracted from non-T2DM networks for sub-network generation.
