# Supplementary material for: Network analysis reveals dysregulated functional patterns in type II diabetic skin
Source: Sci Rep. 2022 Apr 27;12:6889. doi: 10.1038/s41598-022-10652-8 (PMC9046425; doi:10.1038/s41598-022-10652-8)
Supplement: Supplementary file 3 — Supplementary Legends. [file 41598_2022_10652_MOESM3_ESM.pdf]

Network Analysis Reveals Dysregulated Functional Patterns in Type II Diabetic Skin

Chunan Liu[1], Sudha Ram[2], and Bonnie L. Hurwitz[1]*
[1] BIO5 Institute and Department of Biosystems Engineering, University of Arizona, Tucson, AZ, 85721, USA
[2] BIO5 Institute and Department of Management Information Systems, University of Arizona, Tucson, AZ, 85721, USA
* To whom correspondence should be addressed. Phone: 520-626-9819; Email: bhurwitz@email.arizona.edu

Table Legends for Supplementary Tables S1-S8

Supplementary Table S1. Data Summary

Supplementary Table S2. DE Genes in T2DM Skin Samples

Supplementary Table S3. MM, GS, and BC Values for the Genes in the lightgreen Module

Supplementary Table S4. MM, GS, and BC Values for the Genes in the magenta Module

Supplementary Table S5. BC Values for lightgreen Genes in non-T2DM and T2DM Network

Supplementary Table S6. BC Values for magenta Genes in non-T2DM and T2DM Network

Supplementary Table S7. Enrichment Result for the lightgreen Module

Supplementary Table S8. Enrichment Result for the magenta Module
